# The epidemiology and economic burden of hip fractures in Israel

**DOI:** 10.1186/s13584-018-0235-y

**Published:** 2018-08-02

**Authors:** Royi Barnea, Yossi Weiss, Ifat Abadi-Korek, Joshua Shemer

**Affiliations:** 1 Assuta Health Services Research Institute, 20 HaBarzel st, 69710 Tel-Aviv, Israel; 2 The Department of Health Systems Management, Ariel University, Ariel, Israel; 3 Department of Academy and Research, Assuta Medical Center, Tel-Aviv, Israel; 4 Assuta Medical Center Network, Ariel, Israel; 5 Israeli Center for Technology Assessment in Health Care, Gertner Institute, Sheba Medical Center, Tel Hashomer, 52621 Ariel, Israel; 6 Sackler School of Medicine, Tel Aviv University, P.O. Box 39040, 6997801 Tel Aviv, Israel

**Keywords:** Hip fracture, Economic evaluation, Elderly, Rehabilitation, Healthcare costs

## Abstract

**Background:**

Hip fractures increase the risks of mortality and major morbidity in the elderly. Hip fractures are associated with chronic pain, reduced mobility, disability and increasing dependence. We evaluated the direct costs incurred to the Israeli healthcare system in 2013 as a result of hip fracture injuries in elderly patients.

**Methods:**

Hip fractures costs evaluation consisted of first-year and long-term direct costs. Data on the incidence of hip fractures resulting in hospitalizations were retrieved from the Israeli Ministry of Health’s (MOH) Central Database of Hospital Admissions. Hospitalization, rehabilitation and nursing utilization rates and costs were estimated based on the professional literature and according to the MOH’s price list.

**Results:**

During 2013, 6285 elderly patients were hospitalized in Israel due to hip fractures. Direct costs of hip fracture, comprising hospitalization, rehabilitation and nursing costs incurred during the first year after the injury, were estimated at 454 million New Israeli Shekels (NIS; 83,841 NIS per person). Long-term nursing care costs in 2013 were 265 million NIS, with an average cost of approximately 49,000 NIS for 1600 elderly persons receiving long-term nursing care as a result of a hip fracture. Overall, the total direct costs of hip fracture in the elderly population in Israel in 2013 were 719 million NIS.

**Conclusions:**

The direct costs of hip fractures in Israel among the elderly are approximately 719 million NIS per year. The majority of costs are associated with the first year following the injury. To reduce healthcare costs in Israel, changes in the country’s healthcare policy on hip fractures are required. For example, there is a need for a program for detecting high- risk populations, and for early intervention following the injury.

**Electronic supplementary material:**

The online version of this article (10.1186/s13584-018-0235-y) contains supplementary material, which is available to authorized users.

## Background

The hip joint comprises the acetabulum and the femoral head. The femoral neck connects the femoral head to the proximal portion of the femoral shaft and attaches to the intertrochanteric region. The term “hip fracture” applies to fractures in any of these locations [1].

Hip fracture prognosis varies by anatomic location. The intertrochanteric region contains a large amount of cancellous bone with a good blood supply; therefore intertrochanteric fractures typically heal well if reduction and fixation are properly performed. The femoral neck, on the other hand, has only a limited amount of cancellous bone, a thin periosteum, and relatively poor blood supply that can be disrupted by injury. Fractures in the femoral neck area have a higher incidence of complications [1].

Marked variation in hip fracture rates is observed among countries, with a 10-fold variance in hip fracture incidence [2]. The incidence of hip fracture increases exponentially with age in both genders, with most hip fractures occurring in the elderly [3]. Between 1990 and 2000, the peak number of hip fractures for both males and females occurred at 75–79 years of age [4]. Within countries, the age-standardized incidence of hip fractures in men was approximately half that noted in women [2]. In Israel, between 1998 and 2001, the estimated annual incidence of hip fractures in individuals above 50 years of age was 402/100,000 women and 196/100,00 men [5]. Because of the increasing number of elderly people in the world, the total number of hip fractures in individuals 50 years and older will continue to rise and the total number of hip fractures is expected to surpass 6 million by the year 2050 [6, 7].

The major risk factors for hip fractures among elderly individuals are osteoporosis and falls [8–10]. Hip fractures substantially increase the risk of mortality and major morbidity in the elderly [11, 12]. This type of injury is associated with chronic pain, reduced mobility, disability, and an increasing degree of dependence [13], with 40% of patients unable to walk independently, 50% unable to regain their ability to live independently and up to 60% requiring assistance a year later [14–16]. As a result, a large proportion of these patients become completely dependent [17] and often require long term nursing care [18, 19] and admission to a nursing home [19, 20].

Women sustain hip fractures more often than men due to their higher rates of osteoporosis [21]; however, the mortality risk in men is higher than in women [22, 23]. Being a male, a nursing home resident, over 90 years of age, having other comorbidities, and inability to ambulate independently all contribute to mortality risk [24, 25]. The one-year mortality rates of individuals who had a hip fracture range from 17 to 27% and the mortality risk is three-fold higher than that in the general population [26, 27]. Such individuals also have a higher 5-year mortality risk [28].

Understanding the incidence and postsurgical outcomes of hip fractures is a vital first step in improving population health. The aim of this study was to describe the epidemiology of hip fractures in elderly patients (> 65 years) in Israel and to estimate their direct costs to the healthcare system.

## Methods

### Data sources

Data regarding the incidence of hospitalizations as a result of hip fracture, length of stay and demographic characteristics of individuals who sustained hip fracture injuries in Israel in 2013 were retrieved from the Central Registry of Hospital Admissions, which is managed by the Israeli Ministry of Health (MOH).

### Cost estimation

Hip fracture-related costs were based on the model described by Hernlund et al., [29] . In this model, a distinction is made between direct and indirect costs and between first-year and long-term costs. In this study, we calculated only the direct costs, including both direct costs incurred in the first year after the injury and direct costs incurred in the long term.

The assessment of the direct costs during the first year after hip fracture injury included hospitalization costs, rehabilitation costs and nursing costs. Utilization estimates for the the last two were based on the professional literature and unit prices were taken from the MOH’s price list.

#### Hospitalization costs

In estimating the hospitalization costs we combined data on the number and distribution of admissions for each type of hip replacement surgery from the MOH’s Central Registry of Hospital Admissions with unit cost data from the MOH’s price list for hip replacement surgery (Table 1) [30], and the MOH’s price list for hospitalization in general hospitals [30]., on the average length of stay during hospitalization obtained from the Central Registry of Hospital Admissions, and.

**Table 1:** Ministry of Health price list of hospitalization and rehabilitation (and items’ unit costs) following hip fracture injuries

	Price, (New Israeli Shekels)
Hip fracture fixation using plate or in-marrow nailing within 48 hours of hospitalization	27,928
Partial hip replacement, within 48 hours of Hospitalization	52,812
Full hip replacement, within 48 hours of Hospitalization	58,780
General hospital hospitalization , up to three days	2,885 per day
General hospital hospitalization fourth day and onwards	2,509 per day
Geriatrics rehabilitation in general hospital	1,520 per day
Geriatrics rehabilitation in geriatric hospitals	1,206 per day
Rehabilitation department, general hospital	2,509 per day
Rehabilitative care in home setting	450 per hour
Home-care therapist	70 per hour

#### Rehabilitation costs

Rehabilitation costs were estimated by combining estimates based on the professional literature of the percentage of patients who underwent inpatient and/or ambulatory rehabilitation after hip fracture injury [31, 32], with Israel-specific data on the average length of stay and per diem for inpatient rehabilitation (Table 1) [30].

#### Community nursing costs

Community nursing costs were estimated from the National Insurance Institute’s report, which provided information on the number of individuals who were eligible for a nursing benefit [33]. The number of hours required for home nursing after a hip fracture injury was estimated based on publications that reported the percentage of patients who required community nursing after hip fracture injuries [34–36].

#### Long-term nursing costs

Long-term costs mostly include nursing costs for patients who did not require nursing care before becoming injured. The premise is that once an individual requires nursing care due to a hip fracture, he/she will remain under such care for the rest of his/her life. To that end, we estimated the number of patients who required nursing following hip fracture injuries from the MOH registry and excluded those patients who required nursing prior to the injury. The percentage of elderly patients who lived at home prior to suffering a hip fracture injury and were admitted to nursing homes or geriatric institutes following the injury was based on data reported in the literature [31, 35–38].

## Results

### Estimation of the direct costs of hip fracture during the first year after the injury

**Fig. 1:**
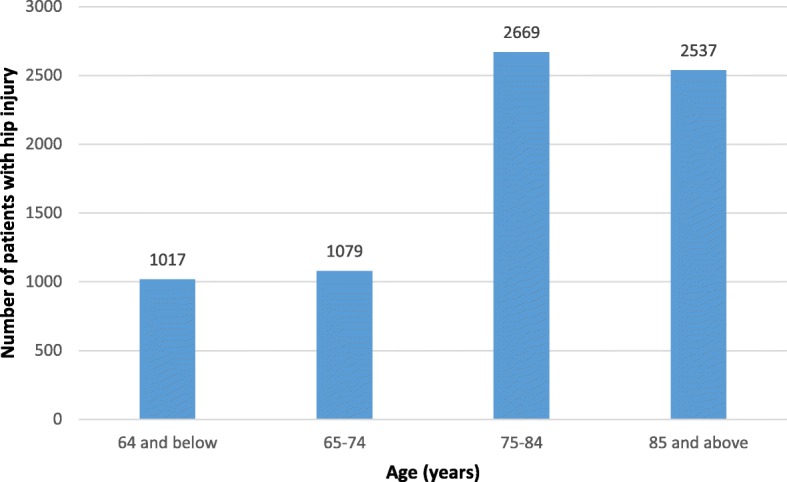
The distribution of patients with hip fracture injury by age

During 2013, 7300 patients, most of whom were above 65 years of age (6284 patients, 86.1%), were hospitalized in Israel due to hip fractures (Fig. 1). More than two-thirds of hip fractures in patients over 65 years occurred in women (4288 patients, 68.2%). Most hip fracture admissions (6649 cases of all ages, 91.1%) were in orthopedic departments of general inpatient institutions, while 643 admissions were in internal medicine departments. The average duration of hospitalization after hip fracture injuries was 7.9 days in orthopedic departments and 9.2 days in internal departments. Of the patients above 65 years of age who had hip fracture injuries, most (5415/6284, 86.2%) underwent surgical operations, which included fixations (64.9% of patients), partial joint replacements (30.6%) and full joint replacements (4.5%).

The direct costs of hip fracture comprised the costs of hospitalization, rehabilitation and nursing costs incurred during the first year after the injury and estimated as described below.

#### Hospitalization costs

According to MOH data, a total of 5415 patients underwent hip replacement surgeries following hip fracture injuries in 2013. Given the average duration of hospitalization following hip fracture injuries, and the number and distribution of surgeries by type, we estimated that hospitalization costs were approximately 277 million New Israeli Shekels (NIS) per year. Notably, in most cases the health maintenance organizations (HMOs) pay medical centers lower fees than those specified in the MOH’s price list. In 2012 the average discount that HMOs received from medical centers was 17% [30, 39], and this was taken into account in our calculations.

#### Rehabilitation costs

**Fig. 2:**
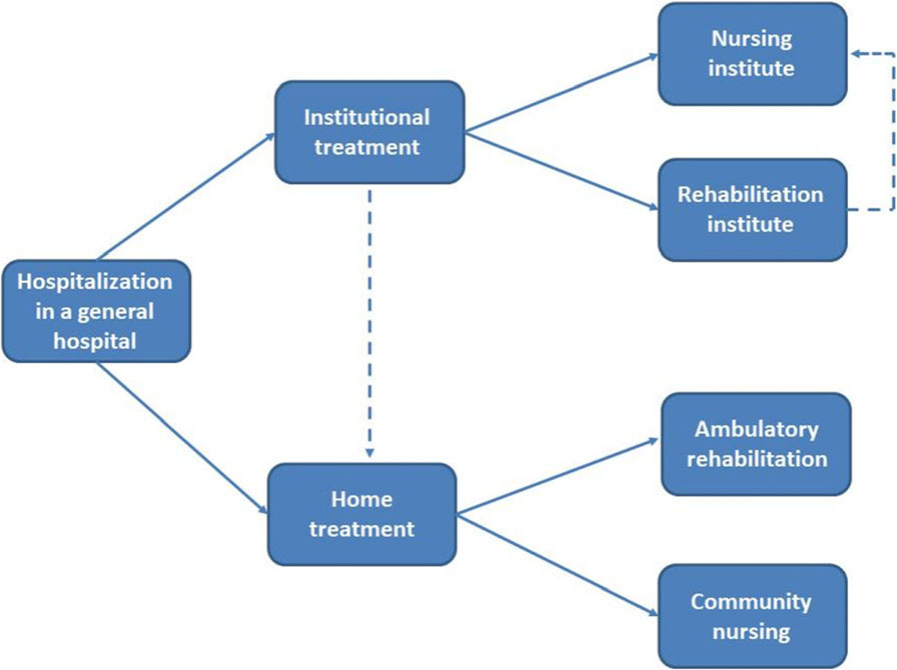
The rehabilitation process following hip fracture injury

In Israel, elderly patients may receive full inpatient rehabilitation in rehabilitation departments of general hospitals, rehabilitation hospitals, chronic disease hospitals, geriatric nursing institutions or geriatric centers. Alternatively, outpatient rehabilitation may be performed in outpatient rehabilitation centers of general hospitals. Ambulatory rehabilitation may be provided in the patient’s home or in outpatient centers operated by HMOs (Fig. 2).

According to data from The Chaim Sheba Medical Center, approximately 60% of elderly individuals who had a hip fracture injury are suitable for rehabilitation [32]. Zucker et al. [31] reported that in 2009–2010, of the 672 patients > 65 years with hip fracture injury who were discharged with recommendations for rehabilitation, 494 (73.5%) received inpatient rehabilitation, 432 (64%) received both inpatient rehabilitation and ambulatory rehabilitation, 96 (14.3%) received ambulatory rehabilitation treatment without an inpatient phase and 82 (12.2%) did not receive any rehabilitation.

Based on these data, we estimated that out of 6284 hip fracture injury cases reported in 2013, 3770 patients (60%) received rehabilitative care. Of these, we estimated that 460 patients (12%) did not receive any rehabilitative treatment, 2770 (74%) received full inpatient rehabilitation, and 2420 (64%) received ambulatory rehabilitation.

##### Inpatient rehabilitation costs

According to the MOH’s registry, in 2013 there were 935 admissions to geriatric rehabilitation departments with an average length of stay of 31.2 days, and 100 admissions to general rehabilitation departments following hip fracture injuries with an average length of stay of 34.2 days. Based on our estimation above that 2770 patients received full inpatient rehabilitation, we postulated that 1735 patients were treated in other rehabilitation institutions but were not registered in the MOH registry. Given the average length of stay for inpatient rehabilitation and hospitalization costs according to the MOH’s price list (Table 1), we estimated that hip fracture inpatient rehabilitation costs are approximately 93 million NIS per year. As mentioned above, HMOs usually pay lower fees than those specified in the HOM’s price list [30, 39].

##### Ambulatory rehabilitation

As reported by Zucker et al. [31], in 2009–2010 approximately 50% of elderly patients with hip fracture injuries received inpatient as well as ambulatory rehabilitation, and 14% received ambulatory rehabilitation only. Therefore, we estimated that in 2013 about 2420 (64%) of patients who had hip fracture injuries received ambulatory rehabilitation. The vast majority of ambulatory rehabilitation (95%) is provided in the home, while 3% are provided in rehabilitation institutes and 2% in outpatient clinics [31]. The most common rehabilitative treatment given to elderly patients recovering from hip fracture injury is physiotherapy provided once weekly [31]. More than 60% of patients require treatment for up to 30 days, 27.9% require treatment for 31 to 60 days and 11.9% of patients require treatment for more than 60 days [31]. From these data, we assumed that most patients received 5 to 6 ambulatory physiotherapy treatments. According to information provided by one of the HMOs, rehabilitative care in home setting costs 450 NIS per treatment. Based on all of the above, we estimated that the cost of ambulatory care following hip fracture injury in elderly individuals is approximately 6 million NIS per year.

#### Community nursing costs

As Israel does not have a national registry that integrates the eligibility of elderly people suffering from hip fracture and their need for community nursing services, we estimated the number of hours required for home nursing during the first year after hip fracture injury from the literature. According to Eilat-Tsanani et al. [35], who examined a population of 91 elderlies with hip fractures, during the first three months after the injury there was a median increase of approximately 40 h of assistance per week. Dimai et al. [34] examined over 14,000 elderly patients with hip fractures and estimated the number of nursing care hours as 83 h per patient. The information from Dimai’s et al. study together with the data on the number of weekly hours allocated to individuals eligible for nursing benefits, has led us to the estimation that elderly patients with hip fracture injuries require community nursing services for a period of 6–10 months. According to Marques et al. [36] 62% of patients who returned to living in the community after hip fracture surgery required home care support or a home-care therapist.

According to Israeli law, an individual who has reached retirement age may be eligible for a geriatric nursing benefit from the National Insurance Institute of Israel if he/she need help performing daily activities or require supervision at home. The entitlement to the nursing benefit and the compensation received depend on the level of supervision or help required by the individual. The nursing benefit covers services such as home nursing, treatments in day centers for the elderly, and provision of various supplies. Based on the National Insurance Institute’s report on the number of individuals who were eligible for the nursing benefit, we estimated that the number of hours allocated to individuals eligible for home nursing was 9.5 a week. The average value of the geriatric nursing benefit in Israel was 2800 NIS per month [33].

All of the above led us to estimate that 65% of patients (3480 patients) discharged from the hospital after hip fracture injury would require 9.5 h per week of community-based nursing for about 8 months, which would cost 78 million NIS.

In summary, the direct costs for hip fracture injuries in elderly patients during the first year after the injury were estimated at 454 million NIS (Table 2).

**Table 2:** Direct costs of hip fracture injuries

Component	Cost (Million New Israeli Shekels)
Hospitalization	277.0
Rehabilitation	
Hospital	93.0
Community based rehabilitation	6.0
Community based nursing	78.0
Total	454.0 (83,841 NIS per person)

#### Long-term direct costs of hip fracture

After sustaining hip fracture injuries, many patients become dependent and require nursing for the rest of their lives [40]. In order to evaluate long-term direct costs, we estimated the number of patients who required long-term nursing following hip fracture injuries and excluded those patients who required nursing prior to the injury.

According to the MOH’s registry and based on our calculations regarding the incidence rate of hip fractures in elderly patients in Israel, currently there are more than 10,900 elderly persons in Israel who had hip fracture injuries (either recently or further in the past). Studies performed in Israel reported that 12.2–19% of elderly patients who lived at home prior to their hip fracture injury, were admitted to nursing homes or geriatric institutes following the injury [31, 35, 37]. Similar percentages were reported for elderly patients in Portugal [36] and in Canada [38] (18 and 20%, respectively). Based on this information, we postulated that 15% of Israeli elderly patients with hip fracture injuries (1635 patients) would require long-term nursing care.

According to the MOH’s price list (Table 1), the cost of a nursing bed is 12,000–15,000 NIS per month which is 144,000–180,000 NIS per year. This cost is subsidized partly by the MOH, subject to income tests conducted for the elderly and their relatives. Elderly persons who are not eligible for funding from the MOH must fund a nursing bed privately [41]. Given the above (1635 patients and 162,000 NIS on average per patient per year), nursing hospitalization costs caused by hip fractures (excluding the first year after the fracture) were estimated by us as 265 million NIS per year.

#### Total direct cost

Overall, the total direct costs of hip fractures in the elderly population in Israel in 2013 were 719 million NIS.

## Discussion

Hip fracture injuries remain a significant health and financial burden despite recently declining fracture rates in many countries. Increases in the absolute number of hip fractures over time reflect the impact of growing populations and increased age in driving an increased burden. This study estimated the direct costs in 2013 of hip fracture injuries in the elderly population in Israel. According to our economic evaluation, first year direct costs of hip fracture injuries, consisting of hospitalization, rehabilitation and nursing costs during the first year after the injury were 454 million NIS, i.e., 83,841 NIS per hip replacement case (21,000 US dollars according to the average exchange rate in 2013). Long-term nursing care costs were 265 million NIS per year. Overall, the total direct costs of hip fracture in the elderly population in Israel in 2013 were 719 million NIS.

**Fig. 3:**
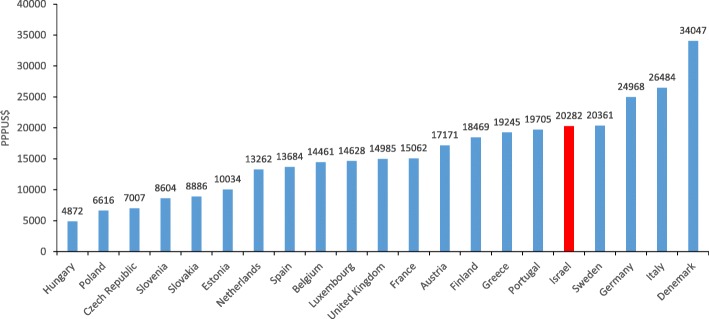
Comparison of direct costs of hip injury in various countries. Data are presented in PPP exchange rates

The total annual hospital costs for 2012/2013 associated with all incident hip fractures in the UK amongst patients above 60 years were estimated at £1.1 billion (£14,264 per incident hip fracture) [42]. The mean 1-year cost of hip fracture for patients aged 50 year and over, who were admitted to an acute care facility in Canada for hip fracture in 1995/1996 was 26,527 Canadian dollars. These costs were significantly different for patients who returned to the community ($21,385), versus those who were transferred to ($44,156), or readmitted to long-term care facilities ($33,729) [43]. We compared the direct costs of hip fractures in the first year following the fracture, which were calculated above (454 million NIS), with the direct first year costs (excluding nursing costs) of hip fractures in the European Union. To compare costs across countries we used market exchange rates for tradeable goods (such as pharmaceuticals and medical equipment) and purchasing power parities (PPPs) for non-tradeable goods (such as professionals) and assumed that 80% of direct costs are for non-tradeable goods. After using these conversion factors, the direct costs of hip fractures in Israel was 14,963 Euros (PPPUS$20,282), which was higher than ….. (Fig. 3), but similar to the costs in Western Europe (14,429 Euros, PPPUS$19,387). (An alternate approach, without discerning between tradeable and non-tradable components, and using PPPs as the conversion factors for all costs, resulted in a similar trend and is presented in Additional file 1: Figure S1).

Notably, it is possible that the estimated direct costs for Israel were somewhat underestimated compared to the estimated direct costs of some of the other countries examined. This difference is derived from additional costs that are related to the direct costs of hip fracture injury including medications, medical aids and doctor visits (which were included in the calculations for other countries but not in the calculations for Israel). However, we believe that our assessment and evaluation is still valid since these costs are often quite small relative to hospitalization and rehabilitation costs.

Examination of the basic demographics of the study’s population presents a similar pattern to the known characteristics of hip fracture worldwide: most of the elderly patients in Israel who suffered from hip fracture were above 65 years old (86%) and more than two-thirds of hip fractures occurred in women. These two findings are consisted with previous studies that have described hip fracture injuries characteristics [3, 5, 21, 44]. Several studies found that inpatient treatment for men compared with women was more expensive [45–47], suggesting that men who experience hip fractures usually have a poor health status, potentially leading to complicated or prolonged hospital stays.

With the growing population of the elderly, prevention may be the best approach for reducing hip fracture costs and consequently healthcare system costs, due to several reasons: first, there is a high incidence of falls among the elderly population (one out of three individuals will fall in a year [5]); second, falls are a leading risk factor for mortality risk among the elderly [26, 27]; third, 20–30% of falls will result in severe damage (hip fracture or head injury [1]) requiring hospitalization (which increases with age); fourth, it was previously postulated that the aging of the population will result in increased hip fracture incidence [6, 7]; and finally, hip fracture as a result of falling leads to functional impairment, dependency and decreased quality of life [13]. Current hip fracture prevention strategies are based on approaches with limited success that may require a long period of time before becoming effective [48]. Bearing in mind the enormous expenses that were presented in the study, we believe that falls prevention (and consequently hip fractures prevention) or at least reducing the incidence of falls should be a top priority. A national program for falls prevention was recently launched by Israel’s Ministry of Health in order to reduce healthcare expenditures and improve the quality of life of elderly individuals. In addition, to face the challenges of early prevention in osteoporotic patients at high risk of hip fracture, surgical intervention should be considered [48].

Timing of the intervention following a hip injury may also lower the costs of hip fractures. In a study conducted in the United States, an early intervention model in which patients underwent an operative intervention less than 6 hours after admission was compared to a late operative intervention that was performed more than 6 hours after admission. The results demonstrated a shorter length of stay and significantly lower costs in the early intervention group. There was no significant difference in the incidence of major complications between the two groups [49]. Therefore, early interventions when treating hip fractures have the potential for larger healthcare savings. Another study showed that delays in time from admission until surgical treatment increased the mortality rate of patients with hip fractures [50]. In Israel, early intervention (< 48 h) constitutes one the quality measures which are being used by the MOH. Furthermore, in 2004, the Israel MOH decided that hospitals would receive the full DRG payment for hip fractures operations only in cases in which the operation is performed within 48 h of hospitalization [51]. Nevertheless, no significant improvement was observed in clinical outcomes, possibly due to other characteristics of the patients (age, co-morbidities, etc.) [51]. Altogether, it could be argued that early surgical intervention seems to play an important role in lowering costs, reducing mortality and achieving shorter recovery.

This study has several limitations, as follows:

1. Israel does not have a national registry that tracks the eligibility of elderly people suffering from hip fracture and their need for community nursing services. Thus, our data were collected from the Central Registry of Hospital Admissions, which is managed by the Israeli MOH, and information from published papers.
2. We used local list prices, based on the maximum allowed prices set by the MOH. These prices are subject to discounts (through negotiations between hospitals and HMOs) as well as to payment caps. Therefore, we may have overestimated the actual cost; for example, In 2012 the average discount that HMOs received from medical centers was 17% [30, 39].
3. We did not include additional costs that are related to the direct costs of hip fracture injury including medications, medical aids and doctor visits.
4. We did not assess the additional impact of co-morbidities on hospitalization costs. Most patients with hip fractures have co-morbidities, which were shown to be directly related to higher hospitalization costs and lengths of stay in hip fracture treatment [46, 52].
5. We did not account for mortality among hip fracture patients.
6. Since 2013 there may have been an increase in the number of patients receiving comprehensive care.
7. This only presents direct costs from the healthcare system perspective. The authors are aware of a wide variety of factors that were not included in the evaluation such as indirect costs (due to loss of quality of life and reduced life expectancy), private expenses on therapists as well as the social-economic burden incurred the patient and his/her family.

Despite these limitations, the authors are confident that the work presented here can promote awareness and a change in Israel healthcare policy regarding hip fracture.

It is important to note that.

## Conclusions

The total (direct) hip fracture costs in Israel are approximately 719 million NIS per year. Since a large portion of falls can be prevented, and due to the high costs of hip fractures, we believe that a change in Israeli healthcare policy regarding hip fractures is required. Such a change may include raising awareness to fall prevention among high-risk populations by increasing information accessibility; identifying high-risk elderly and promoting various interventions (physical and social); reducing risk factors (e.g., environment hazards), implementing integrated care in elderly patients who fell, and initiating an annual layout in order to prevent falls that lead to hip fractures among the elderly. Moreover, early interventions following hip fracture injuries have the potential for larger healthcare savings and should be considered routinely. Finally, we believe that establishing a national registry for hip fracture reports should be a top priority in the MOH’s work plan.

## Additional file

Additional file 1: **Figure S1.** Comparison of direct costs of hip injury in various countries. Data are presented in PPP exchange rates. An alternate approach, without the discerning between tradeable and non-tradable components was performed. This calculation resulted in similar trend. Namely, The direct costs in Israel were relatively high compared to the European countries. (PPTX 114 kb)
