# Supplementary material for: The epidemiology and economic burden of hip fractures in Israel
Source: Isr J Health Policy Res. 2018 Aug 2;7:38. doi: 10.1186/s13584-018-0235-y (PMC6090924; doi:10.1186/s13584-018-0235-y)
Supplement: Supplementary file 1 — Figure S1. Comparison of direct costs of hip injury in various countries. Data are presented in PPP exchange rates. An alternate approach, without the discerning between tradeable and non-tradable components was performed. This calculation resulted in similar trend. Namely, The direct costs in Israel were relatively high compared to the European countries. (PPTX 114 kb) [file 13584_2018_235_MOESM1_ESM.pptx]

## Slide 1
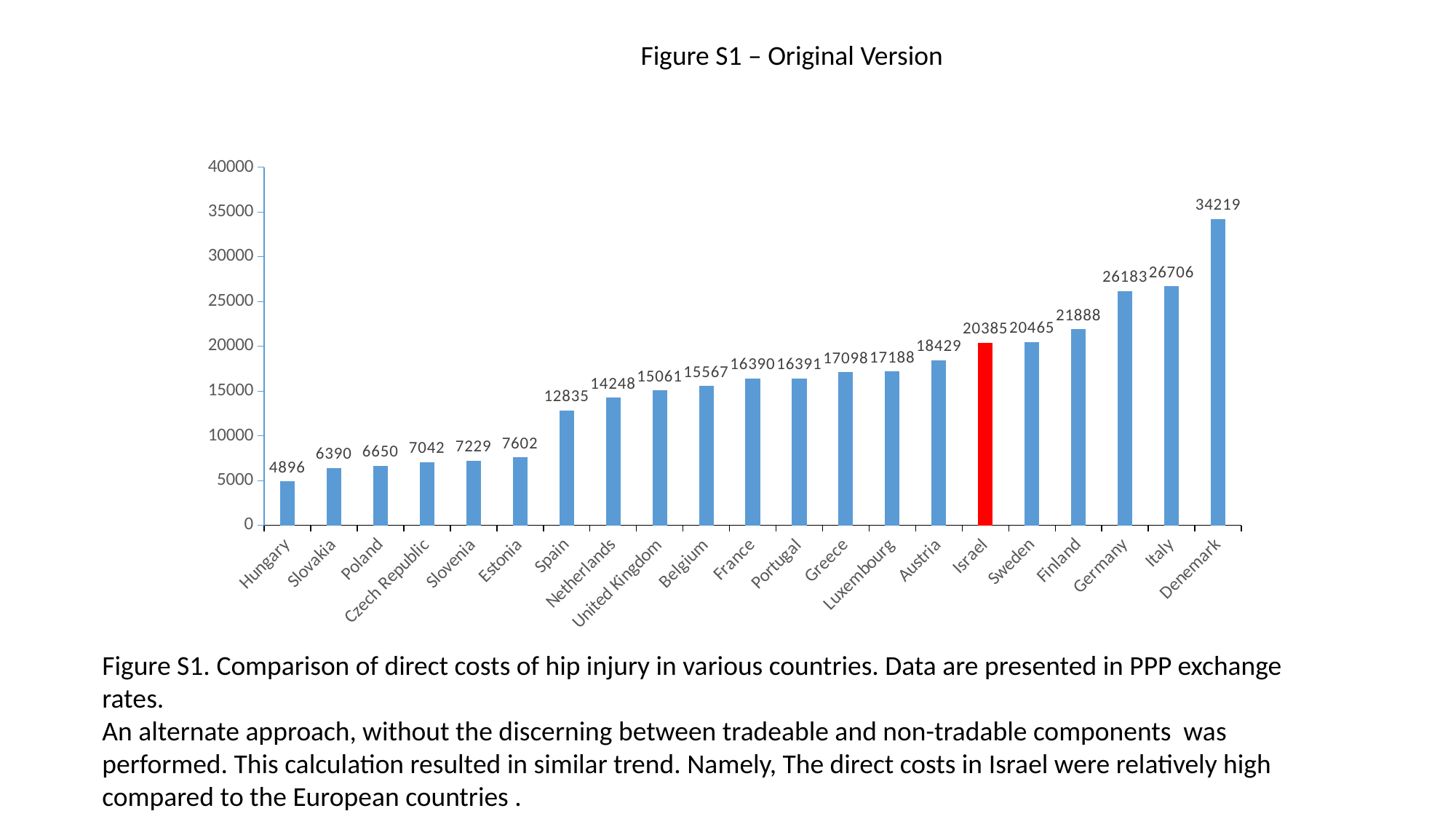

Figure S1 – Original Version
### Chart
| Category | |
|---|---|
| Hungary | 4896.457765667575 |
| Slovakia | 6389.645776566757 |
| Poland | 6649.863760217983 |
| Czech Republic | 7042.234332425068 |
| Slovenia | 7228.882833787466 |
| Estonia | 7602.179836512261 |
| Spain | 12835.149863760218 |
| Netherlands | 14247.956403269754 |
| United Kingdom | 15061.307901907358 |
| Belgium | 15566.757493188012 |
| France | 16389.64577656676 |
| Portugal | 16391.00817438692 |
| Greece | 17098.092643051772 |
| Luxembourg | 17188.010899182562 |
| Austria | 18429.1553133515 |
| Israel | 20385.0 |
| Sweden | 20464.57765667575 |
| Finland | 21888.283378746593 |
| Germany | 26182.56130790191 |
| Italy | 26705.722070844688 |
| Denemark | 34219.346049046326 |Figure S1. Comparison of direct costs of hip injury in various countries. Data are presented in PPP exchange rates.
An alternate approach, without the discerning between tradeable and non-tradable components was performed. This calculation resulted in similar trend. Namely, The direct costs in Israel were relatively high compared to the European countries .
